# A Correlative Study Between IVIM-DWI Parameters and the Expression Levels of Ang-2 and TKT in Hepatocellular Carcinoma

**DOI:** 10.3389/fonc.2020.594366

**Published:** 2021-01-15

**Authors:** Jing Zheng, Xue Qin Gong, Yun Yun Tao, Ran Wang, Gang Yang, Jing Dong Li, Tian Ren, Zu Mao Li, Cui Yang, Wei Cheng Wang, Lin Yang, Xiao Ming Zhang

**Affiliations:** ^1^ Medical Imaging Key Laboratory of Sichuan Province, Department of Radiology, Affiliated Hospital of North Sichuan Medical College, Nanchong, China; ^2^ Institute of Hepato-Biliary-Intestinal Disease, Department of Hepatobiliary Surgery, Affiliated Hospital of North Sichuan Medical College, Nanchong, China; ^3^ Department of Medical Record Statistics, Affiliated Hospital of North Sichuan Medical College, Nanchong, China; ^4^ Department of Pathology, Affiliated Hospital of North Sichuan Medical College, Nanchong, China

**Keywords:** hepatocellular carcinoma, intravoxel incoherent motion, diffusion-weighted imaging, angiopoietin-2, transketolase

## Abstract

**Background:**

Noninvasive evaluation of the expression of angiopoietin-2 (Ang-2) and transketolase (TKT) in hepatocellular carcinoma (HCC) is of great significance for the clinical development of individualized treatment plans. However, the correlation between intravoxel incoherent motion diffusion weighted imaging (IVIM-DWI) and the expression of Ang-2 and TKT has not been reported. We sought to investigate the correlations between IVIM-DWI parameters and Ang-2 and TKT expression levels in HCCs.

**Methods:**

Conventional non-enhanced magnetic resonance imaging (MRI) and IVIM-DWI and dynamic contrast MRI were performed for 61 patients with HCC before surgical treatment. Various IVIM-DWI parameters, such as apparent diffusion coefficient (ADC), slow apparent diffusion coefficient (D), fast apparent diffusion coefficient (D^*^) and fraction of fast apparent diffusion coefficient (f), were calculated using Function-MADC software. Expression levels of Ang-2 and TKT in HCC were detected *via* immunohistochemical staining and classified into two grades. Independent sample *t* tests were used to compare differences in parameters between the two groups. The Spearman rank correlation test was used to analyze the correlations between IVIM-DWI parameters and Ang-2 and TKT expression levels in HCCs.

**Results:**

The D^*^ and f values were significantly higher in the high Ang-2 group than in the low Ang-2 group; there were no obvious between-group differences in ADC and D. Ang-2 expression was positively correlated with D* and f but not with ADC and D. The ADC and D values were significantly lower in the high TKT group than in the low TKT group, whereas the between-group differences for D* and f were not significant. TKT expression was negatively correlated with ADC and D but not with D* and f.

**Conclusions:**

IVIM-DWI can be used to evaluate Ang-2 and TKT expression in HCC.

## Introduction

Hepatocellular carcinoma (HCC) is one of the most common malignant tumors of the digestive system, and it has severe disease burden (1). Traditional imaging methods only show the size and scope of the tumor from the morphological perspective, and they do not reflect water molecule diffusion in tumor tissues. Continuous development of magnetic resonance technology has promoted the use of diffusion-weighted imaging (DWI)-based functional magnetic resonance imaging (MRI) in tumor diagnosis and treatment. However, the apparent diffusion coefficient (ADC) measured using DWI reflects water molecule diffusion and capillary perfusion but not the diffusion of pure water molecules in tissues. Intravoxel incoherent motion (IVIM)-DWI proposed by Le BihanD et al. (2, 3) uses a bi-exponential model to obtain multiple parameters, including D, D*, and f, which distinguish the diffusion of water molecules from the perfusion of capillaries. In recent years, studies have shown the application value of IVIM-DWI in the differential diagnosis of liver cancer (4, 5), histological grading (6–8), and evaluating topical treatment response (9, 10).

Antiangiogenesis therapy for tumors is currently a research hotspot. Angiopoietin (Ang) is an important angiogenic factor. The Ang family members mainly include Ang-1, Ang-2, Ang-3, Ang-4 and angiopoietin-like proteins, among which Ang-2 is strongly expressed and localized predominantly in cancer cells (11). Transketolase (TKT) (12) is one of the key enzymes in the nonoxidative part of the pentose phosphate pathway (PPP), and PPP provides approximately 85% of the pentose sugar required for DNA synthesis in tumor cells (13). Studies have shown that TKT promotes tumor cell proliferation and invasion, which are related to tumor prognosis and recurrence (14–17).

Noninvasive evaluation of the expression of Ang-2 and TKT in HCC is of great significance for clinical development of individualized treatment plans. However, the correlation between IVIM-DWI and the expression of Ang-2 and TKT has not been reported. Therefore, the present study investigated the correlation between IVIM-DWI parameters and the expression of Ang-2 and TKT in HCC tissues to noninvasively evaluate the expression of HCC Ang-2 and TKT, as well as to provide reference information for HCC antiangiogenesis targeted therapy.

## Materials and Methods

### Study Population

We enrolled 61 cases of HCC that received surgical resection in our hospital from January 2018 to September 2019. Inclusion criteria: (1) did not receive surgery, topical therapy, radiotherapy, chemotherapy or targeted therapy; (2) lesion diameter was greater than 1 cm; and (3) postoperative pathology confirmed HCC. Exclusion criteria: (1) MRI contraindications; (2) poor image quality; and (3) incomplete data. All patients underwent upper abdominal plain MRI scan, IVIM-DWI and enhanced MRI scan before surgery.

### MRI Scan

The Discovery 750 3.0T superconducting magnetic resonance scanner (GE, USA) was used with a 32-channel phased array surface coil. All study subjects fasted for more than 4 h and received breathing exercises before the MR scan.

Scanning sequence: Breath-hold transverse-plane fat-suppressed T1WI, respiratory-triggered transverse-plane fat-suppressed T2WI and IVIM-DWI scans were conducted (**Table 1**). Nine b-values were selected from the IVIM-DWI sequence (b=0, 20, 50, 100, 150, 200, 400, 800, and 1000). A high-pressure syringe was used to inject the gadolinium‐diethylenetriamine penta‐acetic acid (Gd-DTPA) contrast agent (15–20 ml) through the vein on the back of the hand at an injection speed of 2–2.5 ml/s, and images of the hepatic artery phase, portal phase and equilibrium phase were collected.

**Table 1:** Magnetic resonance (MR) imaging scanning sequences and parameters.

Sequence	TR/TE (ms)	FA(°)	Matrix (mm^2^)	FOV(mm^2^)	ST(mm)
T1WI	4/2	12	260×192	320×320–360×360	2.6
T2WI	2,609/97	110	384×384	320×320–380×380	5
DCE MRI	4/2	12	224×192	320×320–360×360	5
IVIM-DWI	3,529/60.8	90	128×160	340×340–360×360	5

TR, repetition time; TE, echo time; FA, flip angle; FOV, field of view; ST, section thickness; DCE, dynamic contrast-enhanced.

### Data Measurement

Function-MADC software on a GE AW 4.4 Workstation was used to select the slice with the largest solid tumor area on the IVIM-DWI sequence, and a region of interest (ROI) with an area of 70-80 mm^2^ was manually selected, avoiding areas with tumor necrosis, hemorrhage or cystic degeneration. The ADC, D, D* and f pseudocolor maps were generated, and the ADC value, D value, D* value and f value were measured. Each parameter value was measured three times, and the average was calculated.

### Immunohistochemical Detection

The Ang-2 antibody was purchased from Abcam (UK, catalog number ab153934) and was diluted 1:250. The TKT antibody was purchased from Santa Cruz (USA, catalog number SC-390179) and was diluted 1:100. HCC specimens were embedded in paraffin and sectioned continuously at a thickness of 5 μm for Hematoxylin-Eosin (HE) and immunohistochemical staining. Two physicians separately evaluated the same section. Disagreements were resolved after discussion. For each section, the staining intensities in six high-power fields were recorded. The scoring standards for Ang-2 and TKT protein expression intensity were as described in the literature (11, 12) as follows: 0 points (no staining), one point (weak staining), two points (moderate staining), and three points (strong staining). According to the scoring results, subjects were divided into the following two groups: low expression group with scores of one point and below and high expression group with scores of two points and above (18, 19).

### Statistical Analysis

SPSS22.0 software was used for data analysis. Testing for normality indicated that the parameter values for IVIM-DWI conformed with normal distributions. The independent sample *t* test was used to compare the differences in parameters between two groups with different expression levels of Ang-2 and TKT. Spearman’s correlation analysis was used to analyze the correlation between IVIM-DWI parameters and the expression of Ang-2 and TKT. P<0.05 was considered significantly different.

## Results

There were 61 cases enrolled in this study, including 55 males and six females aged 29–70 years old with an average age of 50.3 ± 10.4 years. Lesion size ranged from 1.2 to 16.1cm with an average size of 5.1 ± 3.3cm.

**Figure 1:**
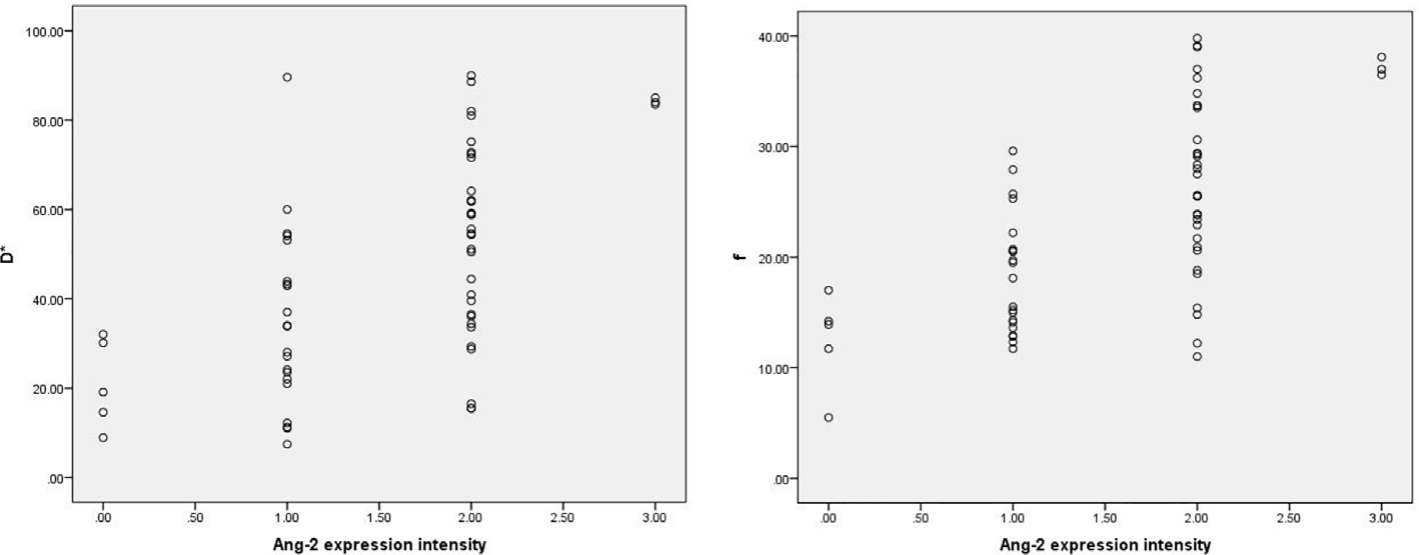
Scatter plots showing that D* and f values were significantly correlated with intensity of Ang-2 expression.. The units for D* values are ×10^-3^mm^2^/s, and the unit for f is %.

**Figure 2:**
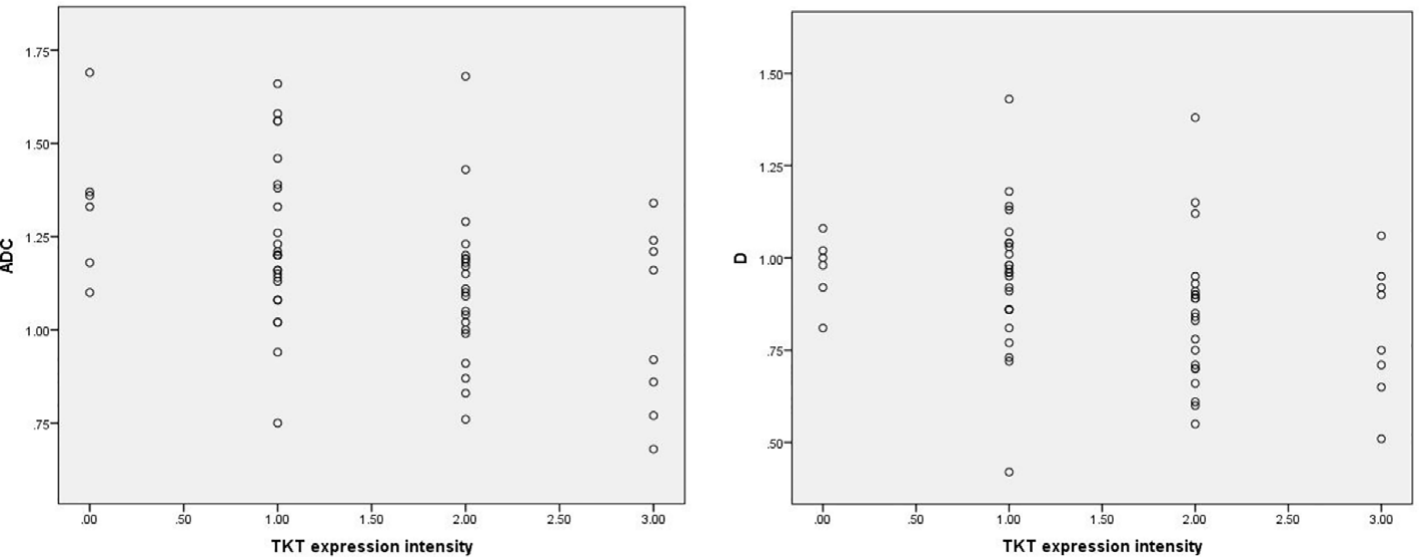
Scatter plots showing that ADC and D values were significantly correlated with intensity of TKT expression. The units for apparent diffusion coefficient (ADC) and D values are ×10^-3^mm^2^/s.

**Figure 3:**
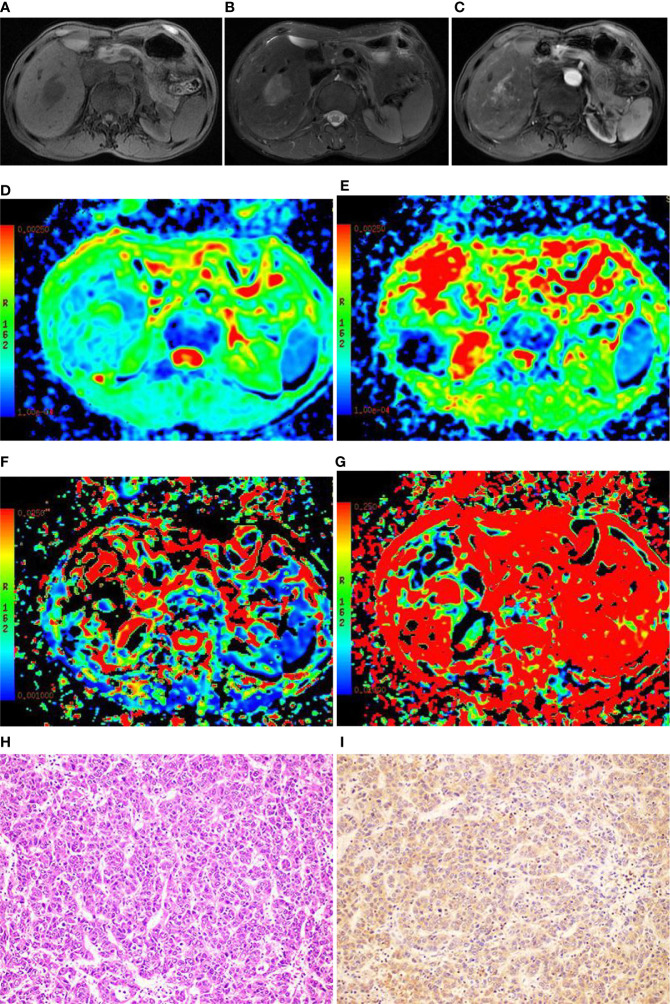
Images of a 49-year-old male with hepatocellular carcinoma (HCC) in the right lobe of the liver. **(A)** T1WI image showing the lesion with slight hyposignal; **(B)** T2WI image showing the lesion with heterogeneous hypersignal; **(C)** magnetic resonance (MR) enhanced scan showing the lesion with obvious heterogeneous enhancement; **(D)** On the apparent diffusion coefficient (ADC) map, the ADC value was 1.21×10^-3^mm^2^/s; **(E)** On the D map, the D value was 1.03×10^-3^mm^2^/s; **(F)** On the D* map, the D* value was 71.6×10^-3^mm^2^/s; **(G)** On the f map, the f value was 33.5%; **(H)** The HE staining (×200) showing that the cancer cells were arranged in a beam-like structure; **(I)** Ang-2 immunohistochemical staining image (×200) showing that Ang-2 was highly expressed.

**Figure 4:**
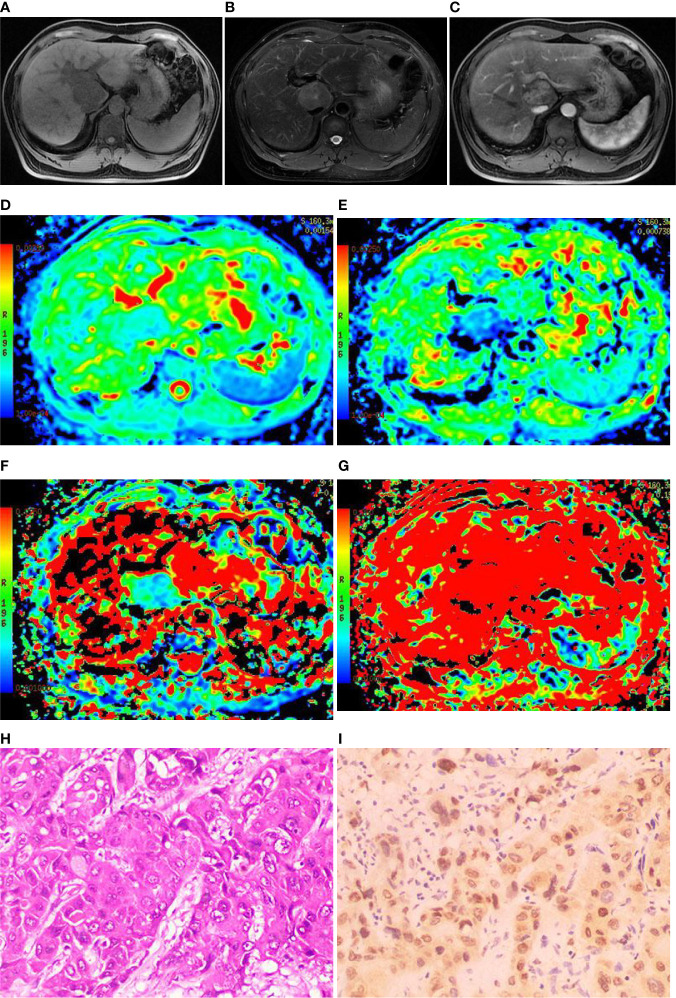
Images of a 44-year-old male with hepatocellular carcinoma (HCC) in hepatic caudal lobe. **(A)** The T1WI image showing the lesion with slight hyposignal; **(B)** The T2WI image showing the lesion with heterogeneous hypersignal; **(C)** The magnetic resonance (MR) enhanced showing the lesion with obvious heterogeneous enhancement; **(D)** On the apparent diffusion coefficient (ADC) map, the ADC value was 1.08×10^-3^mm^2^/s; **(E)** On the D map, the D value was 0.81×10^-3^mm^2^/s; **(F)** On the D* map, the D* value was 24.1×10^-3^mm^2^/s; **(G)** On the f map, the f value was 15.5%; **(H)** Hematoxylin-Eosin (HE) staining (×200) showed that the cancer cells were arranged in a beam-like structure; **(I)** Immunohistochemistry (×200) showed that transketolase (TKT) was highly expressed.

Among the 61 cases of HCC, 35 cases had high expression of Ang-2, and 26 cases had low expression of Ang-2. Moreover, 30 cases had high expression of TKT, and 31 cases had low expression of TKT. The D* and f values were significantly higher for the high Ang-2 expression group (55.71±19.21×10^-3^ mm^2^/s and 27.58±8.09%, respectively) than for the low Ang-2 expression group (32.25±19.22×10^-3^ mm2/s and 17.29±5.66%, respectively), whereas the ADC and D values did not significantly differ between the two groups (1.20±0.22×10^-3^ mm^2^/s and 0.92±0.20×10^-3^ mm^2^/s, respectively, for the high expression group and 1.14±0.24×10^-3^ mm^2^/s and 0.87±0.17×10^-3^ mm^2^/s, respectively, for the low expression group). The D* and f values were positively correlated with Ang-2 expression (r=0.578, p<0.05 and r=0.645, p<0.05, respectively). In contrast, the ADC and D values were not significantly correlated with Ang-2 expression (r=0.132, p>0.05 and r=0.106, p>0.05, respectively). The ADC and D values were significantly lower for the high TKT expression group (1.09±0.21×10^-3^ mm^2^/s and 0.83±0.19×10^-3^ mm^2^/s, respectively) than for the low TKT expression group (1.25±0.21×10^-3^ mm^2^/s and 0.95±0.17×10^-3^ mm^2^/s, respectively), whereas the D* and f values did not significantly differ between the two groups (45.32±23.95×10^-3^ mm^2^/s and 22.22±9.24%, respectively, for the high expression group and 46.09±23.65×10^-3^ mm^2^/s and 24.13±8.31%, respectively, for the low expression group). The ADC and D values were negatively correlated with TKT expression (r=-0.376, p<0.05 and r=-0.386, p<0.05, respectively). In contrast, the D* and f values were not correlated with TKT expression (r=0.040, p>0.05 and r=-0.136, p>0.05, respectively) (**Tables 2**–**4**; **Figures 1**–**4**).

**Table 2:** Comparison of intravoxel incoherent motion diffusion weighted imaging (IVIM-DWI) parameters between groups with different Ang-2 expression.

	Low expression group (n=26)	High expression group (n=35)	*t*	*p*
ADC	1.14 ± 0.24	1.20 ± 0.22	0.968	0.337
D	0.87 ± 0.17	0.92 ± 0.20	0.995	0.324
D^*^	32.25 ± 19.22	55.71 ± 19.21	4.381	0.000
f	17.29 ± 5.66	27.58 ± 8.09	5.549	0.000

The units for apparent diffusion coefficient (ADC), D, and D* values ​​are ×10^-3^mm^2^/s, and the unit for f is %.

**Table 3:** Comparison of intravoxel incoherent motion diffusion weighted imaging (IVIM-DWI) parameters between high and low transketolase (TKT) expression groups.

	Low expression group (n=31)	High expression group (n=30)	*t*	*p*
ADC	1.25 ± 0.21	1.09 ± 0.21	3.023	0.004
D	0.95 ± 0.17	0.83 ± 0.19	2.539	0.014
D^*^	46.09 ± 23.65	45.32 ± 23.95	0.126	0.900
f	24.13 ± 8.31	22.22 ± 9.24	0.852	0.398

The units for apparent diffusion coefficient (ADC), D, and D* values ​​are ×10^-3^mm^2^/s, and the unit for f is %.

**Table 4:** Correlations between intravoxel incoherent motion diffusion weighted imaging (IVIM-DWI) parameters and the expression of Ang-2 and transketolase (TKT).

Spearman correlation	Ang-2		TKT	
	*r*	*p*	*r*	*p*
ADC	0.132	0.312	−0.376	0.003
D	0.106	0.417	−0.386	0.002
D^*^	0.578	0.000	0.040	0.757
f	0.645	0.000	−0.136	0.296

## Discussion

Angiogenesis is closely related to the occurrence and development of tumors (20–23). Ang-2 is highly expressed in HCC tissues (18, 20, 24, 25), and Ang-2 is positively correlated with tumor microvessel density (MVD) (26). The following mechanisms are potential ways in which Ang-2 promotes angiogenesis (27–29): (1) Ang-2 antagonizes the effect of Ang-1 on Tie-2, which inhibits Tie-2 signaling and disrupts signals mediating stable endothelial cell interaction in the Tie-2 signaling pathway, leading to the instability for existing blood vessels; and (2) Ang-2 and VEGF synergistically promote the formation of tumor blood vessels.

Ang-2 is used to monitor tumor response to antiangiogenesis targeted therapy (30). A prior study has shown (31) that a prepared single-chain variable fragment (scFv) Ang-2 single-chain antibody has a significant inhibitory effects on the angiogenesis and tumor growth of HCC *in vivo* and *in vitro*. In addition, specific Ang-2 targeted intervention may act by reshaping the neovascular network and changing the tumor microenvironment. Mueller et al. (32) showed that combined therapy with dual-targeting of Ang-2 and VEGF significantly inhibits tumor activity and that compared to single-targeted therapy of Ang-2 or VEGF, combined therapy has obvious advantages. Moreover, Mueller et al. suggested that antiangiogenesis therapy is helpful for fighting chemotherapy resistance. Previous research on plumbagin inhibition of angiogenesis-mediated tumor growth in HCC has shown (33) that plumbagin inhibits the expression of Ang/Tie2 and has significant antitumor activity, suggesting that plumbagin may be a promising antiangiogenesis drug.

TKT has a high positive expression rate in HCC tissues (16), and TKT enhances the proliferation, migration, invasion and colony formation ability of liver cancer cells (17). Yang et al. (34) showed that TKT protects cervical cancer cells from cisplatin treatment and that targeting TKT may have a therapeutic effect on cervical cancer. Studies on metastatic ovarian cancer have shown (19) that inhibiting TKT expression blocks the proliferation of the SKOV-3 cell line, which is an ovarian cancer cell line, and that oxythiamine, an inhibitor of TKT activity, significantly inhibits the proliferation of four ovarian cancer cell lines and primary serous ovarian cancer cells isolated from the patient’s ascites. Li et al. (35) studied the relationship between TKT expression and bile acid levels in mouse liver cancer tissues. The results showed that TKT is transported to the nuclei of liver cancer cells by interacting with the signal transducer and activator of transcription-1 (STAT-1). TKT then inhibits the expression of the farnesoid receptor (FXR) (a tumor suppressor gene) by promoting the binding of histone deacetylase-3 (HDAC-3) to the FXR promoter, causing increases in intrahepatic bile acid (related to the occurrence of liver cancer). The lack of hepatocyte TKT reduces the level of intrahepatic bile acid, which provides an opportunity for liver cancer treatment. The results of the above studies suggest that TKT may be a novel target for tumor treatment.

Studies have shown that IVIM-DWI noninvasively evaluates tumor angiogenesis (3). Lee et al. (36) studied the relationship between IVIM-DWI parameters of mouse colorectal cancer tissue and MVD, and they reported that the D* and f values are significantly correlated with MVD but that there is no correlation between the MVD and either ADC or D. Lee et al. showed (37) that the value of the IVIM-DWI parameter f is significantly correlated with MVD for HCC and may be used to evaluate the antiangiogenic effect of sorafenib. Wang et al. (38–40) also obtained similar results.

Song et al. (40) divided 25 human gastric cancer-bearing nude mice into a control group and a treatment group, and they performed IVIM-DWI scans. The results showed that the tumor tissue MVD is significantly reduced after chemotherapy and that MVD is positively correlated with the D* and f values of the perfusion-related parameters. Joo et al. used IVIM-DWI imaging technology to quantitatively evaluate the therapeutic effect of vascular disrupting agents on rabbit VX2 liver tumors (41). The results showed that the D* and f values in the treatment group are significantly reduced 4 h after treatment but then recover to baseline at 24 h. In addition, the results demonstrated that the D value significantly increases at 24 h and that the extent of decrease in the f and D* values within 4 h is correlated with the extent of increase in tumor volume measured during the 7-day follow-up after treatment. The above results suggest that the D* and f values may be early predictors for evaluating tumor treatment response. The present study showed that the D* and f values of HCC tissue were significantly correlated with the expression of Ang-2. Higher Ang-2 expression resulted in greater D* and f values and more abundant microcirculation perfusion of HCC tissue, which was consistent with the above results.

Ki-67 promotes the proliferation of cancer cells (42). Surov et al. (43) showed that Ki-67 is negatively correlated with the values ​​of the IVIM-DWI parameters, ADC and D. Wang et al. (44) and Xiao et al. (45) also obtained similar results. The present study demonstrated that the ADC and D values were lower for the high TKT expression group than for the low TKT expression group and indicated that TKT expression was negatively correlated with the ADC and D values. These results may be due to TKT promoting cancer cell proliferation, resulting in limited diffusion of free water molecules, thereby reducing the ADC and D values, which reflect the diffusion of water molecules in the tissue (17, 19).

In recent years, several studies have suggested that TKT has a certain role in promoting angiogenesis, but its specific mechanism is still unclear (16). In our study, there were no differences in the values of the D* and f blood perfusion parameters between the different expression groups of TKT. In future studies, it is necessary to verify the angiogenesis effect of TKT and to explore the correlation between the values of the D* and f blood perfusion parameters of HCC IVIM-DWI and the expression of TKT.

The present study had the following limitations: (1) the sample size was relatively small, and larger sample sizes are needed for subsequent studies; (2) solid tumor regions were selected, and the ROIs were manually delineated, which may have introduced measurement errors (46); and (3) the current selection of b-values was not standardized, which may have impacted the results. For the IVIM-DWI model, at least four different b-values (including b=0) are required to perform double exponential fitting to the signal. Studies (47) have shown that as the value of b gradually decreases, the deviation between the measured parameter value and the reference parameter value continues to increase. At present, most studies use approximately 10 b-values (47–50). Therefore, 9 b-values were used in this study based on referring to the literature. In the future, the standardization of the b number and distribution of b-values should be investigated (4, 47, 48).

## Conclusions

The results of the present study suggested that IVIM-DWI can be used to noninvasively evaluate the expression of Ang-2 and TKT in HCC.

## Data Availability Statement

The original contributions presented in the study are included in the article/supplementary material. Further inquiries can be directed to the corresponding author.

## Author Contributions

JZ and LY wrote the paper. JZ, RW, YYT, XQG, CY, ZML, and WCW collected the data and performed immunohistochemical staining. TR performed statistical analysis. GY and JDL performed surgical resection. XMZ designed the research. All authors contributed to the article and approved the submitted version.

## Funding

This work was supported by Projects of the Department of Science and Technology of Sichuan Province (No. 2016JY0105) and the Department of Education of Sichuan Province (No. 18ZB0222).

## Conflict of Interest

The authors declare that the research was conducted in the absence of any commercial or financial relationships that could be construed as a potential conflict of interest.
